# General and Skin-Specific Health-Related Quality of Life in Patients With Atopic Dermatitis Before and During the COVID-19 Pandemic

**DOI:** 10.1097/DER.0000000000000908

**Published:** 2022-06-08

**Authors:** Kamilla Koszorú, Krisztina Hajdu, Valentin Brodszky, Ákos Szabó, Júlia Borza, Katalin Bodai, Györgyi Pónyai, Andrea Szegedi, Miklós Sárdy, Fanni Rencz

**Affiliations:** From the ∗ Department of Dermatology, Venereology and Dermatooncology, Semmelweis University; † Károly Rácz Doctoral School of Clinical Medicine, Semmelweis University, Budapest; Departments of ‡ Dermatology; § Dermatological Allergology, Faculty of Medicine, University of Debrecen; ∥ Saint Martin Outpatient Center, Pannonhalma; ¶ Department of Health Economics, Corvinus University of Budapest, Hungary; # Department of Dermatology and Allergy, University Hospital, LMU Munich, Germany.

## Abstract

Few studies have investigated health-related quality of life (HRQoL) in patients with atopic dermatitis (AD) during the COVID-19 pandemic.

**Objectives:**

The objectives of this study were to compare HRQoL in adult AD patients before and during the pandemic and to assess measurement performance of 4 HRQoL measures.

**Methods:**

Between 2018 and 2021, a multicenter, cross-sectional survey was conducted, involving 218 adult AD patients. Health-related quality of life outcomes included the EQ-5D-5L, Skindex-16, Dermatology Life Quality Index (DLQI), and DLQI-Relevant (DLQI-R). Severity was measured using objective SCORing Atopic Dermatitis, Eczema Area and Severity Index, and Investigator Global Assessment.

**Results:**

The mean ± SD EQ-5D-5L utility, Skindex-16, DLQI, and DLQI-R scores were 0.82 ± 0.22, 56.84 ± 27.46, 13.44 ± 8.46, and 13.76 ± 8.60, respectively. The patients reported more problems during the pandemic (*P* < 0.05) regarding pain/discomfort (odds ratio [OR], 1.78), worrying (OR, 1.89), concerns about persistence/reoccurrence of disease (OR, 1.88), and social relationships (OR, 1.69). The HRQoL outcomes showed strong correlations with each other (range of *r*_s_, |0.69| to |0.99|). The Skindex-16, DLQI, and DLQI-R were able to discriminate between severity groups with large (η^2^ = 0.20–0.23), whereas the EQ-5D-5L with moderate effect sizes (η^2^ = 0.08–0.11).

**Conclusions:**

Atopic dermatitis patients experienced significantly more problems in some areas of HRQoL during the pandemic. The EQ-5D-5L, Skindex-16, DLQI, and DLQI-R demonstrated good convergent and known-group validity and can be suitable instruments for HRQoL assessment in clinical and research settings.

## CAPSULE SUMMARY

- The pandemic affected health-related quality of life of patients with atopic dermatitis in a few areas, such as pain/discomfort, worrying, concerns about the persistence of skin symptoms, and social relationships.
- The generic measure, EQ-5D-5L, and the dermatology-specific DLQI, DLQI-R, and Skindex-16 demonstrated good convergent and known-group validity across clinical severity groups.

Atopic dermatitis (AD) is a chronic, relapsing, inflammatory skin disease, characterized by the damage of skin barrier together with immunological dysfunctions.^1^ It can develop at any age, but the usual onset is in early childhood and the condition may persist through adulthood or resolve after a few years. The prevalence in the adult population in Europe is 1.2% to 8.7%^2^ and approximately 5% in Hungary.^3^ Persistent itching and scratching may restrict daily functioning, sleeping, ability to work, social interactions, and leisure activities and thus often have a negative effect on patients' health-related quality of life (HRQoL).^4–8^ The COVID-19 pandemic constitutes an additional burden on patients, including the experience of increased anxiety, pessimism, sleep problems, and worsening of symptoms.^9^ Furthermore, the “stay-at-home” order and the fear of getting infected resulted in a decrease in dermatological outpatient visits that could also impact AD patients' lives.^10–12^ Nevertheless, few studies have examined the association of the COVID-19 pandemic and HRQoL outcomes in AD patients.^13–15^

Skin-specific and generic questionnaires are extensively used to measure HRQoL in adult AD patients.^16^ Skin-specific instruments allow comparisons across different skin conditions and are typically more sensitive to HRQoL changes related to the skin disease. Among them, Dermatology Life Quality Index (DLQI) is the most commonly used HRQoL measure in daily practice, treatment guidelines, and patient registries.^5,16,17^ In 2021, the Harmonizing Outcome Measures for Eczema group released a recommendation to uniformly use DLQI as the HRQoL outcome for adult AD patients in clinical trials.^18^ However, measurement properties of the DLQI are not without criticism.^19–24^ A recent study in the United States reported that 55.2% of patients with mild AD mark one or more “not relevant” responses (NRRs) on the DLQI suggesting a content validity problem with the measure.^25^ The Dermatology Life Quality Index–Relevant (DLQI-R) is a recently proposed scoring formula of the DLQI for avoiding possible bias in the NRR option.^26^ Among skin-specific measures, the Skindex instrument family has also been increasingly used in AD; however, few validation studies have been performed with the Skindex-16 in AD.^27–30^

Generic instruments enhance comparisons with nondermatologic diseases or the general population, and some of them enable the estimation of health utilities that can be used to calculate quality-adjusted life years in economic evaluations of treatments.^31^ Recent evidence suggests that the quality of existing economic research in AD, including health utility studies, is insufficient.^32^ The EQ-5D instrument family is the most frequently used approach to obtain health utilities in AD patients.^33^ Moreover, the EQ-5D is the preferred method to obtain health utilities by pharmacoeconomic guidelines in approximately 30 countries, including the United Kingdom, the Netherlands, and Hungary.^34,35^ Although more evidence is available with the earlier version of the instrument (EQ-5D-3L), few AD studies reported utilities on the newer, 5-level version (EQ-5D-5L), with a limited number of them reporting on multiple measurement properties of the instrument.^36–41^

The objective of this study was, therefore, to test measurement properties of commonly used generic and skin-specific HRQoL measures (EQ-5D-5L, Skindex-16, DLQI, DLQI-R) in adult patients with AD using data from a cross-sectional survey carried out in Hungary between 2018 and 2021. Given the COVID-19 outbreak during our data collection period, we further aimed to compare HRQoL of AD patients before and since the start of the pandemic, after controlling for sociodemographic and clinical characteristics.

## METHODS

### Study Design and Patients

A cross-sectional survey was conducted involving 2 academic dermatology clinics (Budapest, Debrecen) and 1 dermatology outpatient clinic (Pannonhalma) in Hungary. Inclusion criteria to the study were as follows: (i) being 18 years or older, (ii) being diagnosed with AD by a dermatologist according to the Hanifin-Rajka criteria, and (iii) signing an informed consent form. The data were collected in 2 waves: “before COVID-19” (March 2018 to March 11, 2020 [ie, the date on which the state of emergency was announced in Hungary]) and “since COVID-19” (June 2020 to January 2021). Note that no patients were recruited to the study from March 11 through May 31, 2020. Ethical permission was granted by the scientific and ethical committee of the Medical Research Council in Hungary (reference no. 29655/2018/EKU). The patients' dermatological examination was performed by physicians trained for usage of clinical severity scales. After taking medical history and doing physical examination, patients were asked to self-complete a questionnaire developed by our research team.

### Questionnaire and Outcome Measures

The questionnaire consisted of 2 parts. The first section, completed by the patient, included the Hungarian versions of the EQ-5D-5L^42,43^; DLQI,^44^ which also allows the use of the DLQI-R scoring^26,45,46^; Skindex-16,^47^ a 0- to 10-point visual analog scale (VAS) for itching and sleep disturbance in the preceding month; a 0- to 10-item patient global assessment (PtGA) VAS for self-reported severity assessment; and a few questions about the history of disease and sociodemographic data. The second section of the questionnaire was completed by dermatologists and included clinical information about comorbidities, treatments, and 3 disease severity measures: Investigator Global Assessment (IGA),^48^ Eczema Area and Severity Index (EASI),^49^ and objective SCORing Atopic Dermatitis (oSCORAD).^50,51^ A detailed description of all HRQoL and disease severity measures is provided in Appendix 1 (Supplemental Digital Content, SDC 1: http://links.lww.com/DER/A118).

### Statistical Analyses

#### Health-Related Quality of Life Impact of COVID-19

Descriptive statistics were used to provide an overview of the sample. We determined the proportion of patients with NRRs on the DLQI. Groups of patients before and since the start of the COVID-19 and those with and without NRRs on DLQI were compared by independent samples *t* test (age and disease duration), Mann-Whitney *U* test (HRQoL and disease severity outcomes), and Fisher exact test (all categorical variables, including ceiling effect). Multivariate linear regressions (HRQoL scale total scores) and ordinal logit regressions (HRQoL item responses) were performed to test whether there is a difference in HRQoL between before and since COVID-19. The regression models were controlled for age, sex, level of education, disease severity (oSCORAD), and type of treatment. In cases when heteroscedasticity was present, we used robust standard errors.

#### Measurement Properties of HRQoL Instruments

Measurement properties were tested in the total sample; however, as a sensitivity analysis, all analyses have been repeated for the “before COVID-19” and “since COVID-19” groups separately. We estimated the proportion of patients who achieved the highest and lowest possible scores on each scale. Ceiling and floor effects were considered present if at least 15% of participants achieved the maximum or minimum score on a given measure.^52^ A substantial ceiling or floor effect in an HRQoL measure is considered a limitation of the instrument because this may lead to the insensitivity of the measure to differentiate between patients with very mild or very severe HRQoL impairment.

To assess convergent validity among HRQoL and severity scales, Spearman correlations were calculated (very weak, *r*_s_ < 0.20; weak, 0.20–0.39; moderate, 0.40–0.60; strong, >0.60).^53^ Strong correlations were expected among skin-specific instruments and moderate correlations between the skin-specific and generic instruments.^36^ Skin-specific questionnaires were also expected to correlate more strongly with disease severity than generic ones.^37^ The Kruskal-Wallis test was used to compare HRQoL scores in the different severity groups. For this, both EASI and oSCORAD scores were categorized according to the cutoff values determined by Chopra et al.^54^ We hypothesized that patients with more severe disease had worse HRQoL. Effect size (ES, η^2^) was computed using the *H* statistic obtained in the Kruskal-Wallis test.^55^ Effect size values were interpreted as follows: small, 0.01 or greater; moderate, 0.06 or greater; and large, 0.14 or greater.^56^ Relative efficiency was determined as the ratio of the ESs of 2 HRQoL instruments, with DLQI as reference. A relative efficiency greater than 1 indicated that the specific HRQoL measure is more efficient than DLQI at discriminating between known severity groups. A *P* value less than 0.05 was considered statistically significant. All statistical analyses were undertaken with SPSS 25.0 (IBM, Armonk, NY) and Stata 14 (StataCorp LP, College Station, TX).

## RESULTS

### Patient Characteristics

A total of 224 AD patients were invited to the study, of whom 218 completed the questionnaire. The mean age was 31.3 years (range, 18–73 years) and 57.8% were women (Table 1). The mean disease duration was 19.0 ± 12.9 years (range, 0–68 years). Overall, 35 patients (16.1%) had dermatologic comorbidities and 194 (89.0%) had nondermatological condition(s). The most frequent comorbidity was allergic rhinitis (59.2%), followed by asthma bronchiale (33.9%) and allergic conjunctivitis (22.9%). Anxiety and depression occurred in 22.0% and 3.7%, respectively. Pollen, dust, and food allergy were present in 48.6%, 36.7%, and 22.5% of the patients, respectively. A total of 63.3%, 23.4%, 2.3%, and 1.4% of the patients received systemic (nonbiological), topical, biological, and phototherapy, respectively. At the time of the survey, 9.6% of the patients were untreated. When comparing patients before and during the pandemic, no significant difference was observed in most sociodemographic characteristics; however, there were small variations in the occurrence of some comorbidities and use of certain treatments (Table 1).

**TABLE 1:** Demographic and Clinical Characteristics of the Patients With Atopic Dermatitis

	Mean (SD) or n (%)			P*
	Total Sample (N = 218)	Before COVID-19 (n = 125)	Since COVID-19† (n = 93)	
Age, y	31.34 (11.68)	31.88 (12.64)	30.61 (10.27)	0.429
Disease duration (missing = 3), y	19.02 (12.91)	18.44 (12.84)	19.80 (13.02)	0.444
Family history of AD	74 (33.9%)	36 (28.8%)	38 (40.9%)	0.082
Sex				
Female	126 (57.8%)	72 (57.6%)	54 (58.1%)	1.000
Male	92 (42.2%)	53 (42.4%)	39 (41.9%)	
Education (missing = 2)				
Primary	12 (5.6%)	8 (6.4%)	4 (4.3%)	0.315
Secondary	112 (51.9%)	68 (54.4%)	44 (47.3%)	
Tertiary	92 (42.6%)	47 (37.6%)	45 (48.4%)	
Employment‡				
Employed full-time	109 (50.0%)	67 (53.6%)	42 (45.2%)	0.273
Employed part time	24 (11.0%)	15 (12.0%)	9 (9.7%)	0.665
Retired or disability pensioner	13 (6.0%)	8 (6.4%)	5 (5.4%)	1.000
Unemployed	12 (5.5%)	8 (6.4%)	4 (4.3%)	0.563
Student	60 (27.5%)	34 (27.2%)	26 (28.0%)	1.000
Other	23 (10.6%)	5 (4.0%)	18 (19.4%)	***0.010***
Nondermatologic comorbidities				
Allergic rhinitis	129 (59.2%)	75 (60.0%)	54 (58.1%)	0.782
Bronchial asthma	74 (33.9%)	51 (40.8%)	23 (24.7%)	0.014
Allergic conjunctivitis	50 (22.9%)	35 (28.0%)	15 (16.1%)	*0.050*
Anxiety	48 (22.0%)	18 (14.4%)	30 (32.3%)	***0.003***
Other nondermatologic conditions	11 (5.0%)	4 (3.2%)	7 (7.5%)	0.105
Sinusitis	8 (3.7%)	6 (4.8%)	2 (2.2%)	0.471
Depression	8 (3.7%)	6 (4.8%)	2 (2.2%)	0.471
Other	58 (26.6%)	28 (22.4%)	30 (32.3%)	***0.042***
Allergies				
Pollen allergy	106 (48.6%)	55 (44.0%)	51 (54.8%)	0.132
Dust allergy	80 (36.7%)	45 (36.0%)	35 (37.6%)	0.887
Food allergy	49 (22.5%)	24 (19.2%)	25 (26.9%)	0.193
Metal allergy	14 (6.4%)	12 (9.6%)	2 (2.2%)	***0.028***
Other allergies	70 (32.1%)	25 (20.0%)	45 (48.4%)	***<0.001***
Current treatment				
None	21 (9.6%)	9 (7.2%)	12 (12.9%)	***0.007***
Solely topical therapy	51 (23.4%)	35 (28.0%)	16 (17.2%)	
Phototherapy	3 (1.4%)	3 (2.4%)	0 (0.0%)	
Systemic nonbiological treatment§	138 (63.3%)	78 (62.4%)	60 (64.5%)	
Biological therapy (dupilumab)	5 (2.3%)	0 (0.0%)	5 (5.4%)	

The bold-italic values refer to the statistically significant difference between the “before COVID-19” and “since COVID-19” groups (P < 0.05).

*Independent samples *t* test or Fisher exact test.

†After March 11, 2020.

‡Multiple responses could be marked.

§Including immunosuppressant, antibiotic, and antiviral treatment in monotherapy or in combination with topical or phototherapy.

AD, atopic dermatitis.

### Disease Severity Outcomes

On the itchiness, sleep disturbance, and PtGA, the VAS mean scores were 7.0 ± 2.9, 5.5 ± 3.5, and 6.0 ± 2.7, respectively (Table 2). Disease severity assessed by the IGA scale yielded a mean of 2.8 ± 1.0, whereas the mean oSCORAD and EASI scores were 35.9 ± 14.6 and 15.8 ± 12.0. The proportion of patients with severe AD was 22.1% with EASI, 45.6% with oSCORAD, and 21.1% according to IGA. There was no significant difference in most severity scores before and since COVID-19, except for the slightly lower oSCORAD scores in the latter group.

**TABLE 2:** Disease Severity and HRQoL Scores of the AD Patients

Outcome Measures*		Total Sample (N = 218)						Before COVID-19 (n = 125)		Since COVID-19 (n = 93)†		
		Mean (SD)	Median (IQR)	Minimum	Maximum	Floor Effect, n (%)	Ceiling Effect, n (%)	Mean (SD)	Median (IQR)	Mean (SD)	Median (IQR)	*P*‡
DLQI (0–30)		13.44 (8.46)	14.00 (6.00–20.00)	0	30	9 (4.1%)	3 (1.4%)	13.57 (8.56)	15.00 (6.00–20.00)	13.28 (8.36)	13.00 (6.00–19.50)	0.801
DLQI-R (0–30)		13.76 (8.60)	14.44 (6.00–21.00)	0	30	9 (4.1%)	3 (1.4%)	13.85 (8.68)	15.00 (6.00–21.00)	13.64 (8.53)	14.00 (6.00–21.00)	0.867
Skindex-16 (0–100)										
Symptoms subscale		62.44 (29.64)	68.75 (37.50–87.50)	0	100	4 (1.8%)	33 (15.1%)	60.87 (30.90)	66.67 (33.33–89.59)	64.56 (27.89)	70.83 (45.83–87.50)	0.465
Emotions subscale		61.21 (29.18)	69.05 (40.48–85.71)	0	100	6 (2.8%)	13 (6.0%)	60.88 (30.28)	69.05 (40.48–88.10)	61.65 (27.80)	69.05 (42.86–85.71)	0.945
Functioning subscale		46.87 (31.48)	46.67 (20.00–74.17)	0	100	22 (10.1%)	10 (4.6%)	47.49 (32.30)	50.00 (16.67–78.34)	46.02 (30.49)	43.33 (20.00–73.33)	0.786
Total score		56.84 (27.46)	61.49 (35.64–80.04)	0	100	3 (1.4%)	3 (1.4%)	56.41 (28.90)	63.33 (31.99–81.65)	57.41 (25.54)	60.79 (40.32–78.52)	0.975
EQ-5D-5L utility (−0.848 to 1)		0.82 (0.22)	0.89 (0.78–0.97)	−0.357	1.000	0 (0%)	49 (22.5%)	0.83 (0.21)	0.89 (0.76–1.00)	0.82 (0.23)	0.88 (0.79–0.96)	0.455
EQ VAS (0–100, missing = 1)		69.15 (20.50)	75.00 (57.00–85.00)	0	100	1 (0.5%)	6 (2.8%)	69.11 (21.28)	75.00 (55.50–85.00)	69.19 (19.54)	75.00 (58.50–85.00)	0.797
Itchiness VAS (1-mo average, 0–10, missing = 1)		7.01 (2.92)	8.00 (5.00–9.00)	0	10	6 (2.8%)	51 (23.4%)	6.75 (3.13)	8.00 (4.00–9.00)	7.36 (2.58)	8.00 (6.00–10.00)	0.285
Sleep disturbance VAS (1-mo average, 0–10, missing = 3)		5.51 (3.53)	6.00 (2.00–9.00)	0	10	25 (11.5%)	36 (16.5%)	5.30 (3.52)	6.00 (2.00–8.00)	5.79 (3.55)	6.50 (2.00–9.00)	0.280
PtGA VAS (0–10, missing = 1)		6.04 (2.74)	7.00 (4.00–8.00)	0	10	7 (3.2%)	21 (9.7%)	5.97 (2.94)	7.00 (4.00–8.00)	6.14 (2.45)	6.50 (4.00–8.00)	0.967
oSCORAD (0–83)		35.91 (14.61)	36.90 (26.60–46.73)	0	71.10	2 (0.9%)	0 (0%)	34.36 (24.48)	31.00 (16.00–48.75)	27.89 (20.79)	23.00 (12.25–41.50)	***0.029***
EASI (0–72)		15.76 (11.99)	14.40 (6.10–21.98)	0	59.40	4 (1.8%)	0 (0%)	17.16 (12.64)	16.55 (6.20–23.75)	13.88 (10.84)	12.00 (6.10–20.25)	0.063
IGA scale (0–5)		2.77 (1.04)	3.00 (2.00–3.00)	0	5	5 (2.3%)	5 (2.3%)	2.80 (1.10)	3.00 (2.00–4.00)	2.73 (0.96)	3.00 (2.00–3.00)	0.362

*Higher scores represent better health status for the EQ VAS and EQ-5D-5L utility and worse health status for all other measures.

†After March 11, 2020.

‡Mann-Whitney *U* test.

AD, atopic dermatitis; DLQI, Dermatology Life Quality Index; DLQI-R, Dermatology Life Quality Index-Relevant; EASI, Eczema Area and Severity Index; HRQoL, health-related quality of life; IGA, Investigator Global Assessment; IQR, interquartile range; oSCORAD, Objective component of Scoring Atopic Dermatitis; PtGA, patient global assessment; VAS, visual analog scale.

### Health-Related Quality of Life Outcomes

**Figure 1:**
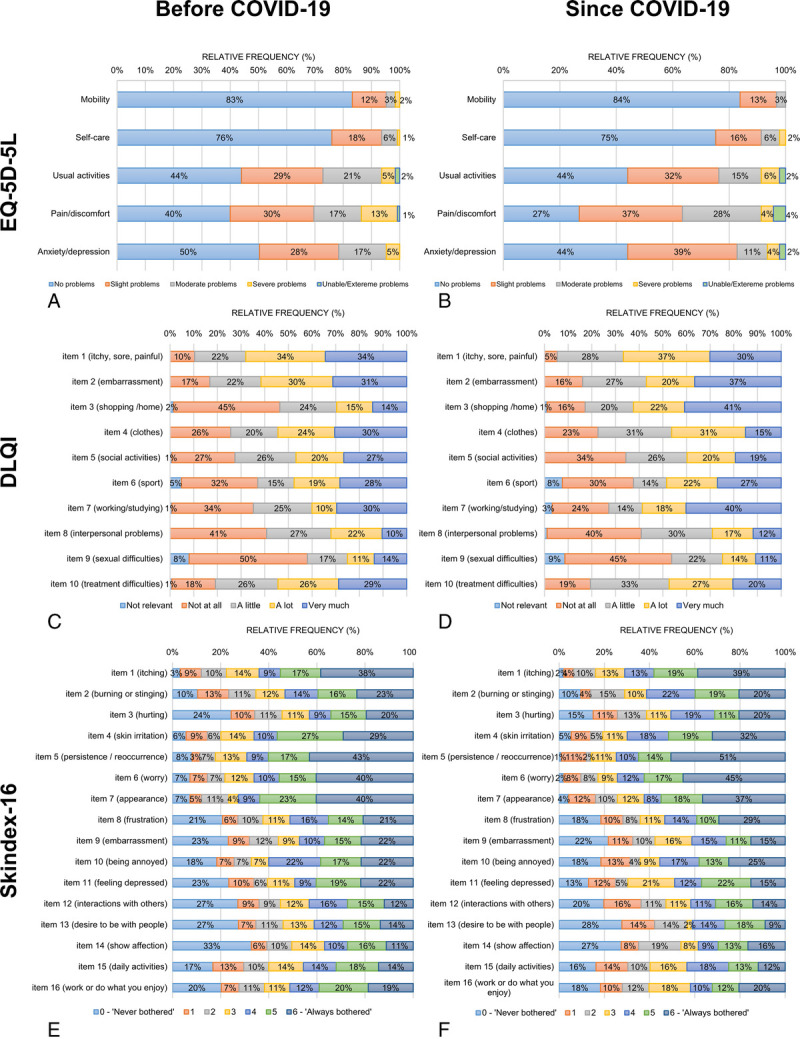
Distribution of responses on the EQ-5D-5L, DLQI, and Skindex-16 before and since COVID-19. A, The EQ-5D-5L before COVID-19. B, The EQ-5D-5L since COVID-19. C, The DLQI before COVID-19. There was one missing response in the item 6 (sport) and another in item 9 (sexual difficulties). D, The DLQI since COVID-19. There was one missing response in the item 7 (working/studying). E, The Skindex-16 before COVID-19. There was one missing response in item 8 (frustration), item 9 (embarrassment), item 10 (being annoyed), item 11 (feeling depressed), and item 15 (daily activities). There were 2 missing responses in item 5 (persistence/reoccurrence), item 12 (interactions with others), and item 14 (show affection). There were 3 missing responses in item 13 (desire to be with people). F, Skindex-16 since COVID-19. There was 1 missing response in item 2 (burning or stinging), item 4 (skin irritation), item 6 (worry), item 7 (appearance), item 11 (feeling depressed), and item 13 (desire to be with people). There were 2 missing responses in item 3 (hurting), item 5 (persistence/reoccurrence), and item 16 (work or do what you enjoy). Percentages may not total 100 because of rounding. “Since COVID-19” refers to after March 11, 2020.

Table 2 presents HRQoL outcomes in the total sample as well as in subsets of patients before and since the pandemic. No significant difference was found in total HRQoL scores before and since COVID-19 as measured by the EQ-5D-5L, EQ VAS, DLQI, DLQI-R, and Skindex-16. However, more patients had problems in some specific areas since COVID-19 including pain/discomfort (60.0% vs 73.1%) and anxiety/depression (49.6% vs 55.9%) on the EQ-5D-5L, shopping/home/garden (53.6% vs 82.8%) and working/studying (64.8% vs 72.8%) on the DLQI, hurting (75.6% vs 84.9%), persistence/reoccurrence of AD (91.9% vs 98.9%), worry (92.7% vs 97.8%), and interactions with others (72.8% vs 80.0%) on the Skindex-16 (Figs. 1A–F). After controlling for sociodemographic and clinical variables, the patients have reported more problems with pain/discomfort (odds ratio [OR], 1.78), shopping/home/garden (OR, 1.86), hurting (OR, 1.87), persistence/reoccurrence of AD (OR, 1.88), worrying (OR, 1.89), and interactions with others (OR, 1.69) since the start of COVID-19 (*P* < 0.05; eTable 1).

In the total sample, 30 patients (13.8%) had at least 1 NRR on the DLQI: 21 (9.6%) 1 NRR, 7 (3.2%) 2 NRRs, and 2 (0.9%) 3 NRRs. The highest number of NRRs were present in items 9 (sexual difficulties), 6 (sports), and 7 (work/school). No significant difference was found between patients with and without NRRs in terms of age, sex, level of education, and disease severity. The patients who were not employed at the time of the survey more often had 1 or more NRRs (*P* = 0.043). When comparing the “before COVID-19” and “since COVID-19” groups, somewhat more NRRs occurred in the latter group (12.1% vs 16.0%, *P* = 0.429). The largest differences were observed in items 6 (sport, 4.8% vs 7.5%, *P* = 0.288) and 7 (work/school, 0.8% vs 4.3%, *P* = 0.106).

### Measurement Properties

No floor effect but a mild ceiling effect was found for the EQ-5D-5L utility, where 22.5% achieved the maximum score (Table 2). No ceiling or floor effects were present for the other HRQoL measures.

We were able to confirm most of our hypotheses regarding convergent validity. The EQ-5D-5L utilities had strong correlations with Skindex-16 total, DLQI, and DLQI-R scores (range of *r*_s_, |0.60| to |0.73|), although only moderate correlations were expected among these measures (Table 3). Skin-specific HRQoL outcomes showed very strong correlations with each other (range of *r*_s_, 0.83 to 0.99). The EQ-5D-5L and EQ VAS correlated weakly (|0.31| to |0.36|), while skin-specific instruments correlated moderately (0.44 to 0.54) with the severity scales (oSCORAD, EASI, and IGA). Itching and sleep disturbance VAS had weak (0.27 to 0.39), whereas PtGA VAS moderate (0.41 to 0.47) correlations with all severity scales. All correlations were statistically significant (*P* < 0.05).

**TABLE 3:** Spearman Correlations Between Outcome Measures (N = 218)

Measures*		DLQI	DLQI-R	Skindex-16				EQ VAS	EQ-5D-5L	Itching VAS	Sleep Disturbance VAS	PtGA VAS	oSCORAD	EASI
				Symptoms Subscale	Emotions Subscale	Functioning Subscale	Total							
DLQI (0–30)		—	—	—	—	—	—	—	—	—	—	—	—	—
DLQI-R (0–30)		0.993	—	—	—	—	—	—	—	—	—	—	—	—
Skindex-16 (0–100)												
Symptoms subscale		0.730	0.725	—	—	—	—	—	—	—	—	—	—	—
Emotions subscale		0.697	0.693	0.722	—	—	—	—	—	—	—	—	—	—
Functioning subscale		0.827	0.822	0.687	0.771	—	—	—	—	—	—	—	—	—
Total		0.839	0.834	0.877	0.904	0.918	—	—	—	—	—	—	—	—
EQ VAS (0–100)		−0.598	−0.592	−0.529	−0.542	−0.591	−0.610	—	—	—	—	—	—	—
EQ-5D-5L (−0.848 to 1)		−0.731	−0.733	−0.572	−0.574	−0.691	−0.684	0.665	—	—	—	—	—	—
Itching VAS (0–10)†		0.579	0.575	0.662	0.583	0.492	0.625	−0.460	−0.452	—	—	—	—	—
Sleep disturbance VAS (0–10)†		0.633	0.632	0.647	0.545	0.535	0.630	−0.459	−0.479	0.726	—	—	—	—
PtGA VAS (0–10)		0.672	0.670	0.682	0.633	0.592	0.695	−0.578	−0.583	0.696	0.620	—	—	—
oSCORAD (0–83)		0.537	0.538	0.461	0.428	0.492	0.516	−0.354	−0.359	0.382	0.385	0.465	—	—
EASI (0–72)		0.485	0.487	0.430	0.384	0.445	0.464	−0.334	−0.308	0.369	0.381	0.409	0.886	—
IGA (0–5)		0.472	0.480	0.377	0.363	0.448	0.443	−0.353	−0.349	0.271	0.320	0.436	0.821	0.809

All correlation coefficients were significant (*P* < 0.05).

*Higher scores represent better health status for the EQ VAS and EQ-5D-5L utility and worse health status for all other measures.

†For the past 1 month.

DLQI, Dermatology Life Quality Index; DLQI-R, Dermatology Life Quality Index-Relevant; EASI, Eczema Area and Severity Index; IGA, Investigator Global Assessment; oSCORAD, Objective component of Scoring Atopic Dermatitis; PtGA, patient global assessment; VAS, visual analog scale.

Consistent with our hypothesis, the patients with more severe disease had worse HRQoL using all outcome measures (Table 4). Skin-specific HRQoL instruments were able to discriminate between known severity groups with large effect sizes (0.20 to 0.23), whereas generic instruments with moderate effect sizes (0.08 to 0.13). The DLQI was able to better distinguish (ie, it showed higher relative efficiency) across 3/3, 2/3, and 1/3 severity groups of the patients than the EQ-5D-5L, Skindex-16, and DLQI-R, respectively.

**TABLE 4:** Known-Group Validity Across the EASI, oSCORAD, and IGA Severity Bands (Mean Scores, Effect Size, Relative Efficiency, N = 218)

EASI Degree of Severity (Missing = 1)		Clear or Mild (0.0–5.9)		Moderate (6–22.9)	Severe (23–72)	Effect Size	Relative Efficiency
n (%)		51 (23.50%)	—	118 (54.38%)	48 (22.12%)	—	—
DLQI (0–30)		8.04 (6.80)	—	13.24 (7.86)	19.46 (7.45)	0.197	—
DLQI-R (0–30)		8.15 (6.91)	—	13.59 (8.02)	19.92 (7.39)	0.204	**1.037**
Skindex-16 (0–100)	Total score	37.14 (26.16)	—	57.84 (25.15)	74.89 (20.37)	0.209	**1.064**
	Symptoms subscale	42.57 (30.69)	—	63.95 (26.52)	80.04 (23.51)	0.174	0.883
	Emotions subscale	42.58 (29.96)	—	62.41 (27.82)	77.33 (19.49)	0.148	0.754
	Functioning subscale	26.28 (27.54)	—	47.18 (29.20)	67.29 (27.14)	0.188	0.957
EQ VAS (0–100)		79.22 (14.95)	—	68.03 (20.87)	61.92 (20.61)	0.086	0.437
EQ-5D-5L (−0.848 to 1)		0.91 (0.14)	—	0.82 (0.21)	0.75 (0.27)	0.080	0.407
oSCORAD degree of severity (missing = 1)		**Clear or mild (0.0–23.9)**		**Moderate (24–37.9)**	**Severe (38–83)**	**Effect size**	**Relative efficiency**
n (%)		45 (20.74%)	—	73 (33.64%)	99 (45.62%)	—	—
DLQI (0–30)		7.07 (6.31)	—	11.95 (7.14)	17.59 (7.97)	0.228	—
DLQI-R (0–30)		7.19 (6.46)	—	12.34 (7.35)	17.94 (8.03)	0.227	0.997
Skindex-16 (0–100)	Total score	36.40 (25.70)	—	52.83 (23.25)	69.66 (23.94)	0.227	0.997
	Symptoms subscale	40.28 (29.33)	—	59.93 (24.70)	75.00 (26.12)	0.198	0.868
	Emotions subscale	44.02 (30.83)	—	56.78 (26.65)	72.90 (24.72)	0.148	0.651
	Functioning subscale	24.89 (28.36)	—	41.78 (27.27)	61.08 (28.62)	0.200	0.879
EQ VAS (0–100)		79.98 (13.15)	—	70.73 (18.43)	62.73 (22.37)	0.091	0.398
EQ-5D-5L (−0.848 to 1)		0.92 (0.11)	—	0.86 (0.18)	0.76 (0.26)	0.089	0.391
IGA		**Clear or almost clear**	**Mild**	**Moderate**	**Severe**	**Effect size**	**Relative efficiency**
n (%)		32 (14.68%)	32 (14.68%)	108 (49.54%)	46 (21.10%)	—	—
DLQI (0–30)		5.44 (5.42)	10.31 (6.70)	14.57 (7.67)	18.54 (8.47)	0.224	—
DLQI-R (0–30)		5.53 (5.56)	10.52 (6.89)	14.93 (7.85)	19.00 (8.34)	0.231	**1.033**
Skindex-16 (0–100)	Total score	26.88 (22.59)	51.53 (21.77)	62.00 (24.85)	69.25 (24.70)	0.215	0.963
	Symptoms subscale	32.81 (30.08)	56.12 (21.92)	68.91 (26.89)	72.28 (26.68)	0.166	0.744
	Emotions subscale	30.43 (26.30)	60.86 (25.51)	65.96 (26.49)	71.69 (25.94)	0.167	0.744
	Functioning subscale	17.40 (21.03)	37.61 (28.05)	51.14 (29.88)	63.77 (28.19)	0.204	0.912
EQ VAS (0–100)		83.53 (12.81)	76.50 (15.98)	65.74 (19.87)	61.93 (23.01)	0.130	0.580
EQ-5D-5L (−0.848–1)		0.92 (0.11)	0.91 (0.08)	0.82 (0.20)	0.71 (0.31)	0.110	0.492

*P* < 0.001 for all groups. Bolded relative efficiency values indicate that the measure is more efficient than DLQI at discriminating between known severity groups.

DLQI, Dermatology Life Quality Index; DLQI-R, Dermatology Life Quality Index Relevant; EASI, Eczema Area and Severity Index; IGA, Investigator Global Assessment; oSCORAD, Objective component of Scoring Atopic Dermatitis; VAS, visual analog scale.

Most measurement properties of the HRQoL instruments were similar in the “before COVID-19” and “since COVID-19” groups, and only a few differences were observed. The ceiling effect of the EQ-5D-5L was lower in the “since COVID-19” group (27.2% vs 16.1%) indicating that fewer patients reported to be in full health since the start of the pandemic (eTable 2). However, ceiling effect for the EQ VAS changed only minimally before and since COVID-19 (3.2% vs 2.2%). In most instances, correlations between the HRQoL measures were slightly stronger before COVID-19 (eTables 3–4). Furthermore, there were some variations in relative efficiency of the measures between the 2 groups (eTables 5–6).

## DISCUSSION

This study is among the first investigations to compare HRQoL in AD patients before and since the start of the COVID-19 pandemic. In addition, this is the first study to concurrently compare measurement properties of the EQ-5D-5L, Skindex-16, DLQI, and DLQI-R in this patient population. The AD patients in our sample indicated quite severe overall HRQoL impairment both before and during the COVID-19 pandemic as attested by the relatively high average DLQI score even in patients with clear or mild AD (DLQI range, 5.4–8.1). The mean EQ-5D-5L utility of 0.82 is similar to values reported in diabetes (0.80)^57^ and partly controlled asthma patients (0.80).^58^ Although no significant decrease in HRQoL was observed with the EQ-5D-5L, Skindex-16, DLQI, and DLQI-R, certain specific problems have become more common among AD patients since the start of the pandemic, including pain/discomfort, worrying, and fear of the persistence/reoccurrence of AD. Moreover, the proportion of patients in full health on the EQ-5D-5L nearly halved during the pandemic. These findings correspond to previous work, where AD patients reported an increased level of anxiety during the pandemic.^9,13^ It is well known that psychosocial stress can negatively impact the course of chronic inflammatory skin diseases, such as AD.^59–61^ The increased problems reported with pain/discomfort and concerns about the persistence or reoccurrence of lesions in our patients may be consequences of the lockdown measures and restricted access to regular outpatient care.

An important finding of our study is that a much smaller proportion of patients provided NRRs (13.8%) on the DLQI compared with what was observed in pemphigus (53.7%),^62^ morphea (36.6%),^62^ hidradenitis suppurativa (20.7%),^63^ vitiligo (76.6%),^64^ psoriasis (22.1%–48.0%),^20,21,26,65,66^ and mild AD (55.2%).^25^ However, in this latter study, the DLQI was completed in an online survey targeting patients with mild disease as reflected in the difference in mean DLQI scores between the 2 studies (4.4 vs 13.8).^25^ Interestingly, we observed that patients marked NRRs slightly more often during the pandemic (12.1% vs 16.0%). Restrictions and lifestyle changes during the pandemic may be responsible for this increase in NRRs, which was also described in psoriasis patients in Ireland,^67^ and our study provides some supportive evidence for this assumption.

All HRQoL measures exhibited good convergent and known-group validity with each other and disease severity scales. Unlike previous studies,^36,68^ where at most, moderate correlations were observed between generic and skin-specific instruments in AD, the EQ-5D-5L and EQ VAS strongly correlated with the Skindex-16, DLQI, and DLQI-R. As expected, the correlation coefficients of severity scores and effect sizes were smaller with the generic EQ-5D-5L than with the 3 skin-specific measures. The differences in performance across the DLQI, DLQI-R, and Skindex-16 were very small in this sample. The performance of the EQ-5D-5L slightly fell short behind that of skin-specific measures. This is very likely because the EQ-5D-5L dimensions are not specific to the symptoms of AD, and therefore, the descriptive system may be less sensitive at detecting slight differences in HRQoL, especially in mild disease.

Our findings have important implications for researchers, clinicians, guideline developers, and decision makers in health care. First, DLQI is the recommended HRQoL assessment tool in AD by the Harmonizing Outcome Measures for Eczema group^18,69^ and clinical guidelines in the United States, the United Kingdom, Japan, Norway, and Singapore.^17,70,71^ The DLQI-R is a quite new initiative; therefore, up to now the problem of NRRs in the DLQI was only highlighted by 1 guideline (German treatment guideline for psoriasis).^72^ Skindex-16, together with the DLQI, is recommended by the Japanese AD treatment guidelines.^73^ Based on our study, Skindex-16, DLQI, and DLQI-R also have good convergent and known-group validity in AD that make them appropriate instruments for HRQoL assessment in both clinical and research settings. Users are suggested to select measure(s) that best suit to their needs, taking into account the differences in content, length, and response scales between DLQI/DLQI-R and Skindex-16.^74^ Moreover, this study also provides EQ-5D-5L utilities stratified according to severity groups (Table 4) that are considered useful as input data in economic evaluations of AD treatments. With the introduction of emerging modern and costly therapies to the treatment of AD, such as the biological drugs (eg, dupilumab) and JAK inhibitors (eg, baricitinib, upadacitinib, abrocitinib), there is growing need for such analyses to inform decision makers and optimize allocation of health resources.^75–77^

This study has a few limitations. First, most participants were enrolled at university hospitals where patients with moderate and severe AD may be overrepresented compared with mild AD. Second, we observed some variation between the before and since COVID-19 groups in the proportion of patients with “other” employment category, the presence of comorbidities, and the current treatment. The latter may be a consequence of the somewhat less frequent outpatient visits since the start of the pandemic and also that the access to dupilumab therapy has improved for adult AD patients in Hungary after 2020. However, in the majority of sociodemographic (age, sex, education) and clinical characteristics (disease duration, family history, most comorbidities) and disease severity scores (PtGA VAS, EASI, and IGA), there was no significant difference between the “before COVID-19” and “since COVID-19” groups. Third, AD-specific HRQoL questionnaires, such as Quality of Life Index for Atopic Dermatitis^78^ or Atopic Dermatitis Burden Scale for Adults (ABS-A),^79^ were not used because of the lack of Hungarian versions. Finally, our study had a cross-sectional design, and longitudinal data (ie, before and since COVID-19) were not available for individual patients that could have allowed a precise analysis of the impact of COVID-19 on HRQoL. Further investigations are, therefore, recommended in this direction.

To conclude, AD patients reported more problems during the pandemic, mostly in the pain/discomfort and mental areas of HRQoL (eg, worry, fear from reoccurrence). All 4 HRQoL outcomes (EQ-5D-5L, DLQI, DLQI-R, and Skindex-16) performed well against validity tests. These HRQoL outcomes may be used as standalone measures or to complement AD-specific HRQoL instruments in clinical trials and daily practice. The EQ-5D-5L results are also suitable to estimate quality-adjusted life years in cost-effectiveness analyses of AD treatments in supporting reimbursement decisions in health care.

## Supplementary Material
