# Supplementary material for: General and Skin-Specific Health-Related Quality of Life in Patients With Atopic Dermatitis Before and During the COVID-19 Pandemic
Source: Dermatitis. 2022 Jun 8;33(6 Suppl):S92–S103. doi: 10.1097/DER.0000000000000908 (PMC9674441; doi:10.1097/DER.0000000000000908)
Supplement: Supplementary file 1 [file der-33-s092-s001.docx]

**Online-only supplementary material**

**Appendix 1:** Description and scoring of HRQoL and disease severity measures

**eTable 1:** Comparison of HRQoL outcomes in AD patients before and since COVID-19 controlled for age, sex, level of education, disease severity (oSCORAD) and type of treatment (N=218)

**eTable 2:** Ceiling and floor effects of outcome measures before and since COVID-19

**eTable 3:** Spearman’s correlations between outcome measures before COVID-19 (N=125)

**eTable 4:** Spearman’s correlations between outcome measures since the start of COVID-19 (N=93)

**eTable 5:** Known-group validity across the EASI, oSCORAD and IGA severity bands before COVID-19 (mean scores, effect size, relative efficiency) [N=125]

**eTable 6:** Known-group validity across the EASI, oSCORAD and IGA severity bands since the start of COVID-19 (mean scores, effect size, relative efficiency) [N=93]

**Appendix 1 Description and scoring of HRQoL and disease severity measures**

***EQ-5D-5L and EQ VAS***

EQ-5D-5L records patients’ HRQoL on the day of completion in five dimensions (mobility, self-care, usual activities, pain/discomfort, and anxiety/depression), and has a five-level response scale in each dimension (1: no problems, 2: slight problems, 3: moderate problems, 4: severe problems and 5: unable to/extreme problems).[^1^](#_ENREF_1) As a result, 5^5^ = 3125 different health states can be described, for which a utility can be assigned, representing societal preferences based on population studies. In this study, we used the Hungarian EQ-5D-5L value set, whereby utilities range from -0.848 to 1 (1 meaning full health, while negative values meaning worse than dead).[^2^](#_ENREF_2) The EQ-5D-5L has a second part, the EQ visual analogue scale (EQ VAS), that is a vertical, 20-cm scale which measures individuals’ perceptions of their current health status from 0 (“the worst health you can imagine”) to 100 (“the best health you can imagine”).

***Dermatology Life Quality Index (DLQI) and DLQI-Relevant (DLQI-R)***

DLQI is the most commonly used skin-specific HRQoL instrument in AD patients.[^3^](#_ENREF_3) It consists of 10 questions, asking about symptoms, feelings, daily activities, leisure, work/school, personal relationships and treatment related to the skin condition. The answer to each item is scored from 0 to 3 (‘not at all’ or ‘not relevant’ = 0, ‘a little’ = 1, ‘a lot’ = 2 and ‘very much’ = 3). The items refer to the preceding seven days and the answers yield a final score of 0-30, where higher scores represent a greater impact on HRQoL.

DLQI-Relevant (DLQI-R) is an alternative scoring of DLQI that adjusts the total score to the number of “not relevant” responses (NRRs) using the following formula:[^4-6^](#_ENREF_4)

DLQI-R$=DLQI\times\frac{10}{10-NRR}$

***Skindex-16***

Like DLQI, Skindex-16 measures the impact of skin condition on HRQoL in the preceding seven days of completion.[^7^](#_ENREF_7) It consists of three subscales (symptoms, emotions, functioning), and altogether 16 questions. Answers are given on a 7-point bipolar rating scale, anchored by ‘never bothered’ and ‘always bothered’, where higher scores mean greater impact on HRQoL. To calculate subscale scores, scores of the corresponding questions are summed up and transformed to a scale of 0-100. A Skindex-16 total score can be calculated by averaging the scores of the three subscales.

***AD severity assessment***

Disease severity was assessed by dermatologists using the following three scoring systems. With Investigator Global Assessment (IGA) as described by Eichenfield et al., physicians evaluated the overall severity of AD on a scale from 0 (clear skin, no inflammatory signs of AD) to 5 (very severe disease).[^8^](#_ENREF_8) Using Eczema Area and Severity Index (EASI), the extent of lesions in four anatomical areas (head/neck, trunk, upper and lower extremities) and the intensity of redness, thickness, excoriation and lichenification in each region were evaluated from 0 to 3.[^9^](#_ENREF_9) The final score lies between 0-72, where higher scores indicate higher severity. SCORing Atopic Dermatitis (SCORAD) consists of three domains.[^10^](#_ENREF_10) First, the involved body surface is calculated according to the Wallace rule of nines. Second, the intensity of the lesions (erythema, edema/papulation, oozing/crust, excoriation, lichenification, dryness) is scored from 0 to 3 each. The third part includes two VAS (0-10) for itching and sleep disturbance. In this study, the objective SCORAD (oSCORAD) was used, which includes only the extent and intensity domains and yields a final score between 0-83, where higher scores indicate higher severity.[^11^](#_ENREF_11)

**eTable 1 Comparison of HRQoL outcomes in AD patients before and since COVID-19 controlled for age, sex, level of education, disease severity (oSCORAD) and type of treatment (N=218)**

| **Item** | **OR (95%CI) or marginal effect (95%CI)** | ***p*-value** |
| --- | --- | --- |
| **EQ-5D-5L** | | |
| dimension 1 (mobility) | 1.26 (0.58-2.74) | 0.564 |
| dimension 2 (self-care) | 1.24 (0.64-2.38) | 0.525 |
| dimension 3 (usual activities) | 1.14 (0.67-1.93) | 0.628 |
| dimension 4 (pain/discomfort) | 1.78 (1.06-2.99) | ***0.028*** |
| dimension 5 (anxiety/depression) | 1.33 (0.78-2.27) | 0.300 |
| EQ-5D-5L utility | -0.03 (-0.09-0.03) | 0.313 |
| EQ VAS | -2.18 (-7.20-2.84) | 0.393 |
| **DLQI** | | |
| item 1 (itchy, sore, painful, stinging) | 1.28 (0.76-2.17) | 0.358 |
| item 2 (embarrassed, self-conscious) | 1.45 (0.85-2.45) | 0.170 |
| item 3 (shopping, home, garden) | 1.86 (1.08-3.20) | ***0.026*** |
| item 4 (clothing) | 0.83 (0.50-1.37) | 0.460 |
| item 5 (social, leisure) | 0.90 (0.54-1.51) | 0.692 |
| item 6 (sport) | 1.23 (0.72-2.09) | 0.445 |
| item 7 (working, studying) | 0.84 (0.50-1.42) | 0.523 |
| item 8 (interpersonal problems) | 1.33 (0.79-2.25) | 0.289 |
| item 9 (sexual difficulties) | 1.55 (0.87-2.77) | 0.141 |
| item 10 (treatment difficulties) | 1.57 (0.94-2.62) | 0.084 |
| DLQI total score | 0.94 (-0.91-2.80) | 0.317 |
| DLQI-R total score | 1.06 (-0.82-2.94) | 0.266 |
| **Skindex-16** | | |
| item 1 (itching) | 1.68 (1.00-2.82) | 0.051 |
| item 2 (burning or stinging) | 1.48 (0.91-2.43) | 0.118 |
| item 3 (hurting) | 1.87 (1.13-3.08) | ***0.015*** |
| item 4 (skin irritation) | 1.48 (0.89-2.45) | 0.134 |
| item 5 (persistence / reoccurrence) | 1.88 (1.09-3.23) | ***0.022*** |
| item 6 (worry) | 1.89 (1.11-3.22) | ***0.019*** |
| item 7 (appearance) | 1.00 (0.60-1.67) | 0.994 |
| item 8 (frustration) | 1.51 (0.91-2.49) | 0.108 |
| item 9 (embarrassment) | 1.17 (0.71-1.93) | 0.540 |
| item 10 (being annoyed) | 1.20 (0.74-1.97) | 0.460 |
| item 11 (feeling depressed) | 1.44 (0.87-2.37) | 0.156 |
| item 12 (interactions with others) | 1.69 (1.03-2.78) | ***0.039*** |
| item 13 (desire to be with people) | 1.16 (0.70-1.92) | 0.568 |
| item 14 (show affection) | 1.59 (0.96-2.63) | 0.072 |
| item 15 (daily activities) | 1.21 (0.74-1.98) | 0.447 |
| item 16 (work or do what you enjoy) | 1.25 (0.76-2.04) | 0.383 |
| Skindex-16 symptoms subscale | 7.41 (0.68-14.14) | ***0.031*** |
| Skindex-16 emotions subscale | 4.66 (-1.88-11.19) | 0.162 |
| Skindex-16 functioning subscale | 3.48 (-3.79-10.75) | 0.345 |
| Skindex-16 total score | 5.18 (-0.78-11.15) | 0.088 |

DLQI = Dermatology Life Quality Index; DLQI-R = Dermatology Life Quality Index Relevant; EQ VAS = EQ visual analogue scale; oSCORAD = Objective component of Scoring Atopic Dermatitis

**eTable 2 Ceiling and floor effects of outcome measures before and since COVID-19**

| **Outcome measures**^a^ | | **Before COVID-19 (N=125)** | | | | **Since COVID-19 (N=93)**^b^ | | | |
| --- | --- | --- | --- | --- | --- | --- | --- | --- | --- |
|  |  | **Minimum** | **Maximum** | **Floor effect, N (%)** | **Ceiling effect, N (%)** | **Minimum** | **Maximum** | **Floor effect, N (%)** | **Ceiling effect, N (%)** |
| **EQ-5D-5L utility (-0.848 to 1)** | | 0.154 | 1.000 | 0 (0.00%) | 34 (27.2%) | -.357 | 1.000 | 0 (0.00%) | 15 (16.1%) |
| **EQ VAS (0-100) (missing=1)** | | 0 | 100 | 1 (0.8%) | 4 (3.2%) | 8 | 100 | 0 (0.0%) | 2 (2.2%) |
| **DLQI (0-30)** | | 0 | 30 | 7 (5.6%) | 3 (2.4%) | 0 | 29 | 2 (2.2%) | 0 (0.0%) |
| **DLQI-R (0-30)** | | 0 | 30 | 7 (5.6%) | 3 (2.4%) | 0 | 29 | 2 (2.2%) | 0 (0.0%) |
| **Skindex-16 (0-100)** | **Total score** | 0 | 100 | 2 (1.6%) | 3 (2.4%) | 0 | 29 | 1 (1.1%) | 0 (0.0%) |
|  | **Symptoms subscale** | 0 | 100 | 2 (1.6%) | 20 (16.0%) | 0 | 98.89 | 2 (2.2%) | 13 (14.0%) |
|  | **Emotions subscale** | 0 | 100 | 5 (4.0%) | 9 (7.2%) | 0 | 100 | 1 (1.1%) | 4 (4.3%) |
|  | **Functioning subscale** | 0 | 100 | 16 (12.8%) | 7 (5.6%) | 0 | 100 | 6 (6.5%) | 3 (3.2%) |
| **Itchiness VAS (1-month average) (0-10) (missing=1)** | | 0 | 10 | 5 (4.0%) | 27 (21.6%) | 0 | 100 | 1 (1.1%) | 24 (25.8%) |
| **Sleeping VAS (1-month average) (0-10) (missing=3)** | | 0 | 10 | 16 (12.8%) | 18 (14.4%) | 0 | 10 | 9 (9.7%) | 18 (19.4%) |
| **PtGA VAS (0-10) (missing=1)** | | 0 | 10 | 7 (5.6%) | 13 (10.4%) | 1 | 10 | 0 (0.0%) | 7 (7.5%) |
| **oSCORAD (0-83)** | | 0 | 69.20 | 1 (0.8%) | 0 (0.0%) | 0 | 71.10 | 1 (1.1%) | 0 (0.0%) |
| **EASI (0-72)** | | 0 | 59.40 | 3 (2.4%) | 0 (0.0%) | 0 | 58.80 | 1 (1.1%) | 0 (0.0%) |
| **IGA scale (0-5)** | | 0 | 5 | 3 (2.4%) | 3 (2.4%) | 0 | 5 | 2 (2.2%) | 2 (2.2%) |

^a^Higher scores represent better health status for the EQ VAS and EQ-5D-5L utility and worse health status for all other measures.

^b^After March 11, 2020.

CE = ceiling effect; DLQI = Dermatology Life Quality Index; DLQI-R = Dermatology Life Quality Index-Relevant; EASI = Eczema Area and Severity Index; FE =floor effect; HRQoL = Health related Quality of Life; IGA = Investigator Global Assessment; IQR = Interquartile range; oSCORAD = Objective component of Scoring Atopic Dermatitis; PtGA = Patient global assessment visual analogue scale; VAS = Visual Analog Scale.

**eTable 3 Spearman’s correlations between outcome measures before COVID-19 (N=125)**

| **Measures**^a^ | | **DLQI** | **DLQI-R** | **Skindex-16** | | | | **EQ VAS** | **EQ-5D-5L** | **Itching VAS** | **Sleep disturbance VAS** | **PtGA VAS** | **oSCORAD** | **EASI** |
| --- | --- | --- | --- | --- | --- | --- | --- | --- | --- | --- | --- | --- | --- | --- |
|  |  |  |  | **Symptoms subscale** | **Emotions subscale** | **Functioning subscale** | **Total** |  |  |  |  |  |  |  |
| **DLQI (0-30)** | | - | - | - | - | - | - | - | - | - | - | - | - | - |
| **DLQI-R (0-30)** | | 0.990 | - | - | - | - | - | - | - | - | - | - | - | - |
| **Skindex-16 (0-100)** | **Symptoms subscale** | 0.770 | 0.771 | - | - | - | - | - | - | - | - | - | - | - |
|  | **Emotions subscale** | 0.713 | 0.715 | 0.755 | - | - | - | - | - | - | - | - | - | - |
|  | **Functioning subscale** | 0.853 | 0.845 | 0.766 | 0.780 | - | - | - | - | - | - | - | - | - |
|  | **Total** | 0.851 | 0.849 | 0.915 | 0.903 | 0.925 | - | - | - | - | - | - | - | - |
| **EQ VAS (0-100)** | | -0.613 | -0.610 | -0.557 | -0.539 | -0.596 | -0.611 | - | - | - | - | - | - | - |
| **EQ-5D-5L (-0.848-1)** | | -0.754 | -0.757 | -0.648 | -0.617 | -0.687 | -0.706 | 0.633 | - | - | - | - | - | - |
| **Itching VAS (0-10)**^b^ | | 0.643 | 0.648 | 0.722 | 0.620 | 0.565 | 0.681 | -0.523 | -0.521 | - | - | - | - | - |
| **Sleep disturbance VAS (0-10)**^b^ | | 0.688 | 0.691 | 0.671 | 0.596 | 0.578 | 0.659 | -0.523 | -0.601 | 0.759 | - | - | - | - |
| **PtGA VAS (0-10)** | | 0.713 | 0.711 | 0.747 | 0.690 | 0.642 | 0.749 | -0.663 | -0.668 | 0.729 | 0.666 | - | - | - |
| **oSCORAD (0-83)** | | 0.542 | 0.542 | 0.527 | 0.476 | 0.491 | 0.549 | -0.327 | -0.363 | 0.416 | 0.454 | 0.442 | - | - |
| **EASI (0-72)** | | 0.498 | 0.495 | 0.481 | 0.454 | 0.460 | 0.501 | -0.316 | -0.288 | 0.415 | 0.427 | 0.378 | 0.858 | - |
| **IGA (0-5)** | | 0.508 | 0.509 | 0.436 | 0.430 | 0.455 | 0.481 | -0.362 | -0.354 | 0.300 | 0.408 | 0.450 | 0.804 | 0.763 |

^a^Higher scores represent better health status for the EQ VAS and EQ-5D-5L utility and worse health status for all other measures.

^b^For the past one month

*p*<0.005 for all groups.

DLQI = Dermatology Life Quality Index; DLQI-R = Dermatology Life Quality Index-Relevant; EASI = Eczema Area and Severity Index; IGA = Investigator Global Assessment; oSCORAD = Objective component of Scoring Atopic Dermatitis; PtGA VAS = Patient global assessment visual analogue scale; VAS = Visual Analogue Scale.

**eTable 4 Spearman’s correlations between outcome measures since the start of COVID-19 (N=93)**

| **Measures**^a^ | | **DLQI** | **DLQI-R** | **Skindex-16** | | | | **EQ VAS** | **EQ-5D-5L** | **Itching VAS** | **Sleep disturbance VAS** | **PtGA VAS** | **oSCORAD** | **EASI** |
| --- | --- | --- | --- | --- | --- | --- | --- | --- | --- | --- | --- | --- | --- | --- |
|  |  |  |  | **Symptoms subscale** | **Emotions subscale** | **Functioning subscale** | **Total** |  |  |  |  |  |  |  |
| **DLQI (0-30)** | | - | - | - | - | - | - | - | - | - | - | - | - | - |
| **DLQI-R (0-30)** | | 0.994 | - | - | - | - | - | - | - | - | - | - | - | - |
| **Skindex-16 (0-100)** | **Symptoms subscale** | 0.679 | 0.665 | - | - | - | - | - | - | - | - | - | - | - |
|  | **Emotions subscale** | 0.672 | 0.661 | 0.675 | - | - | - | - | - | - | - | - | - | - |
|  | **Functioning subscale** | 0.777 | 0.773 | 0.559 | 0.743 | - | - | - | - | - | - | - | - | - |
|  | **Total** | 0.809 | 0.797 | 0.816 | 0.909 | 0.892 | - | - | - | - | - | - | - | - |
| **EQ VAS (0-100)** | | -0.560 | -0.553 | -0.481 | -0.542 | -0.569 | -0.599 | - | - | - | - | - | - | - |
| **EQ-5D-5L (-0.848-1)** | | -0.692 | -0.698 | -0.442 | -0.508 | -0.690 | -0.635 | 0.723 | - | - | - | - | - | - |
| **Itching VAS (0-10)**^b^ | | 0.491 | 0.482 | 0.559 | 0.534 | 0.375 | 0.538 | -0.353 | -0.325 | - | - | - | - | - |
| **Sleep disturbance VAS (0-10)**^b^ | | 0.561 | 0.561 | 0.605 | 0.480 | 0.462 | 0.580 | -0.355 | -0.295 | 0.673 | - | - | - | - |
| **PtGA VAS (0-10)** | | 0.625 | 0.623 | 0.578 | 0.541 | 0.508 | 0.605 | -0.430 | -0.432 | 0.639 | 0.561 | - | - | - |
| **oSCORAD (0-83)** | | 0.520 | 0.523 | 0.407 | 0.336 | 0.476 | 0.462 | -0.389 | -0.368 | 0.346 | 0.318 | 0.496 | - | - |
| **EASI (0-72)** | | 0.462 | 0.472 | 0.395 | 0.265 | 0.413 | 0.407 | -0.352 | -0.347 | 0.309 | 0.337 | 0.450 | 0.914 | - |
| **IGA (0-5)** | | 0.412 | 0.428 | 0.306 | 0.247 | 0.427 | 0.376 | -0.330 | -0.350 | 0.222 | 0.197*** | 0.410 | 0.841 | 0.869 |

‘Since COVID-19’ refers to after March 11, 2020.

^a^Higher scores represent better health status for the EQ VAS and EQ-5D-5L utility and worse health status for all other measures.

^b^For the past one month

*p*<0.05 for all groups except one correlation (Sleep disturbance - VAS-IGA *p*-value was 0.06)

DLQI = Dermatology Life Quality Index; DLQI-R = Dermatology Life Quality Index-Relevant; EASI = Eczema Area and Severity Index; IGA = Investigator Global Assessment; oSCORAD = Objective component of Scoring Atopic Dermatitis; PtGA VAS = Patient global assessment visual analogue scale; VAS = Visual Analogue Scale.

**eTable 5** **Known-group validity across the EASI, oSCORAD and IGA severity bands before COVID-19 (mean scores, effect size, relative efficiency) [N=125]**

| **EASI severity bands**  **(missing = 1)** | | **Clear or mild (0.0-5.9)** |  | **Moderate (6-22.9)** | **Severe (23-72)** | **Effect size** | **Relative efficiency** |
| --- | --- | --- | --- | --- | --- | --- | --- |
| **N (%)** | | 29 (23.4%) | - | 63 (50.8%) | 32 (25.8%) | - | - |
| **DLQI (0-30)** | | 7.10 (6.67) | - | 13.48 (7.67) | 19.25 (7.66) | 0.232 | - |
| **DLQI-R (0-30)** | | 7.19 (6.79) | - | 13.86 (7.86) | 19.54 (7.57) | 0.231 | 0.996 |
| **Skindex-16**  **(0-100)** | **Total score** | 31.28 (26.34) | - | 58.92 (25.89) | 73.63 (21.24) | 0.244 | **1.053** |
|  | **Symptoms subscale** | 35.06 (30.75) | - | 63.82 (26.03) | 78.65 (25.33) | 0.230 | 0.991 |
|  | **Emotions subscale** | 35.22 (28.60) | - | 64.63 (28.47) | 75.67 (19.97) | 0.208 | 0.898 |
|  | **Functioning subscale** | 23.56 (28.11) | - | 48.31 (29.74) | 66.56 (27.30) | 0.201 | 0.869 |
| **EQ VAS (0-100)** | | 80.69 (15.21) | - | 66.54 (22.02) | 64.72 (20.80) | 0.075 | 0.324 |
| **EQ-5D-5L (-0.848-1)** | | 0.91 (0.17) | - | 0.81 (0.22) | 0.81 (0.18) | 0.088 | 0.378 |
| **oSCORAD severity bands (missing = 1)** | | **Clear or mild (0.0-23.9)** |  | **Moderate (24-37.9)** | **Severe (38-83)** | **Effect size** | **Relative efficiency** |
| **N (%)** | | 23 (18.5%) | - | 36 (29.0%) | 65 (52.4%) | - | - |
| **DLQI (0-30)** | | 5.39 (5.18) | - | 12.81 (7.08) | 17.09 (8.10) | 0.247 | - |
| **DLQI-R (0-30)** | | 5.50 (5.40) | - | 13.27 (7.25) | 17.35 (8.16) | 0.242 | 0.978 |
| **Skindex-16**  **(0-100)** | **Total score** | 27.21 (23.55) | - | 53.21 (22.72) | 69.38 (24.75) | 0.294 | **1.191** |
|  | **Symptoms subscale** | 29.53 (27.58) | - | 57.41 (23.21) | 74.81 (26.06) | 0.288 | **1.163** |
|  | **Emotions subscale** | 32.40 (28.09) | - | 59.26 (25.94) | 72.78 (25.35) | 0.230 | 0.930 |
|  | **Functioning subscale** | 19.71 (25.68) | - | 42.96 (28.02) | 60.56 (29.46) | 0.216 | 0.873 |
| **EQ VAS (0-100)** | | 82.70 (12.48) | - | 69.47 (19.16) | 63.62 (22.73) | 0.101 | 0.409 |
| **EQ-5D-5L (-0.848-1)** | | 0.93 (0.11) | - | 0.85 (0.20) | 0.78 (0.23) | 0.103 | 0.416 |
| **IGA** | | **Clear or almost clear** | **Mild** | **Moderate** | **Severe** | **Effect size** | **Relative efficiency** |
| **N (%)** | | 21 (16.8%) | 15 (12.0%) | 57 (45.6%) | 32 (25.6%) | - | - |
| **DLQI (0-30)** | | 4.43 (4.68) | 11.20 (6.36) | 14.88 (7.50) | 18.34 (8.57) | 0.270 | - |
| **DLQI-R (0-30)** | | 4.43 (4.68) | 11.65 (6.67) | 15.22 (7.67) | 18.64 (8.45) | 0.274 | **1.013** |
| **Skindex-16**  **(0-100)** | **Total score** | 22.08 (21.12) | 54.12 (22.96) | 62.25 (25.39) | 69.62 (24.62) | 0.268 | 0.993 |
|  | **Symptoms subscale** | 26.39 (27.81) | 56.39 (22.92) | 67.98 (26.91) | 72.92 (26.77) | 0.227 | 0.839 |
|  | **Emotions subscale** | 24.94 (23.44) | 60.63 (25.61) | 68.00 (26.11) | 71.88 (26.31) | 0.247 | 0.912 |
|  | **Functioning subscale** | 14.92 (20.29) | 45.33 (29.86) | 50.76 (30.58) | 64.06 (28.14) | 0.223 | 0.825 |
| **EQ VAS (0-100)** | | 83.90 (12.89) | 78.87 (17.01) | 64.31 (20.41) | 63.22 (23.40) | 0.151 | 0.557 |
| **EQ-5D-5L (-0.848-1)** | | 0.94 (0.12) | 0.92 (0.07) | 0.81 (0.21) | 0.74 (0.26) | 0.107 | 0.395 |

*p*<0.05 for all groups. Bolded relative efficiency values indicate that the measure is more efficient than DLQI at discriminating between known severity groups.

DLQI = Dermatology Life Quality Index; DLQI-R = Dermatology Life Quality Index Relevant; EASI = Eczema Area and Severity Index; IGA = Investigator Global Assessment; oSCORAD = Objective component of Scoring Atopic Dermatitis; VAS = Visual Analogue Scale.

**eTable 6 Known-group validity across the EASI, oSCORAD and IGA severity bands since the start of COVID-19 (mean scores, effect size, relative efficiency) [N=93]**

| **EASI severity bands**  **(missing = 1)** | | **Clear or mild (0.0-5.9)** |  | **Moderate (6-22.9)** | **Severe (23-72)** | **Effect size** | **Relative efficiency** |
| --- | --- | --- | --- | --- | --- | --- | --- |
| **N (%)** | | 22 (23.7%) | - | 55 (59.1%) | 16 (17.2%) | - | - |
| **DLQI (0-30)** | | 9.27 (6.93) | - | 12.96 (8.13) | 19.88 (7.25) | 0.139 | - |
| **DLQI-R (0-30)** | | 9.41 (7.01) | - | 13.29 (8.27) | 20.69 (7.22) | 0.154 | **1.109** |
| **Skindex-16**  **(0-100)** | **Total score** | 44.86 (24.39) | - | 56.61 (24.45) | 77.41 (18.89) | 0.157 | **1.129** |
|  | **Symptoms subscale** | 52.46 (28.31) | - | 64.09 (27.31) | 82.81 (19.83) | 0.111 | 0.800 |
|  | **Emotions subscale** | 52.27 (29.54) | - | 59.87 (27.10) | 80.65 (18.65) | 0.100 | 0.720 |
|  | **Functioning subscale** | 29.85 (26.99) | - | 45.88 (28.78) | 68.75 (27.65) | 0.141 | **1.013** |
| **EQ VAS (0-100)** | | 77.27 (14.73) | - | 69.71 (19.55) | 56.31 (19.68) | 0.102 | 0.733 |
| **EQ-5D-5L (-0.848-1)** | | 0.90 (0.10) | - | 0.84 (0.19) | 0.65 (0.37) | 0.094 | 0.674 |
| **oSCORAD severity bands (missing = 1)** | | **Clear or mild (0.0-23.9)** |  | **Moderate (24-37.9)** | **Severe (38-83)** | **Effect size** | **Relative efficiency** |
| **N (%)** | | 22 (23.7%) | - | 37 (39.8%) | 34 (36.6%) | - | - |
| **DLQI (0-30)** | | 8.82 (7.00) | - | 11.11 (7.20) | 18.53 (7.74) | 0.212 | - |
| **DLQI-R (0-30)** | | 8.95 (7.09) | - | 11.44 (7.43) | 19.07 (7.77) | 0.215 | **1.015** |
| **Skindex-16**  **(0-100)** | **Total score** | 46.00 (24.78) | - | 52.46 (24.06) | 70.18 (22.66) | 0.147 | 0.696 |
|  | **Symptoms subscale** | 51.51 (27.32) | - | 62.39 (26.14) | 75.37 (26.62 | 0.112 | 0.531 |
|  | **Emotions subscale** | 56.17 (29.36) | - | 54.38 (27.46) | 73.11 (23.84) | 0.085 | 0.400 |
|  | **Functioning subscale** | 30.30 (30.57) | - | 40.63 (26.85) | 62.06 (27.35) | 0.164 | 0.774 |
| **EQ VAS (0-100)** | | 77.14 (13.52) | - | 71.95 (17.88) | 61.06 (21.92) | 0.083 | 0.390 |
| **EQ-5D-5L (-0.848-1)** | | 0.90 (0.10) | - | 0.87 (0.15) | 0.72 (0.32) | 0.072 | 0.340 |
| **IGA** | | **Clear or almost clear** | **Mild** | **Moderate** | **Severe** | **Effect size** | **Relative efficiency** |
| **N (%)** | | 11 (11.8%) | 17 (18.3%) | 51 (54.8%) | 14 (15.1%) | - | - |
| **DLQI (0-30)** | | 7.36 (6.41) | 9.53 (7.07) | 14.24 (7.92) | 19.00 (8.54) | 0.139 | - |
| **DLQI-R (0-30)** | | 7.64 (6.68) | 9.53 (7.07) | 14.61 (8.11) | 19.83 (8.32) | 0.154 | **1.107** |
| **Skindex-16**  **(0-100)** | **Total score** | 36.04 (23.43) | 49.24 (21.09) | 61.73 (24.47) | 68.40 (25.79) | 0.117 | 0.836 |
|  | **Symptoms subscale** | 45.08 (31.72) | 55.88 (21.70) | 69.93 (27.10) | 70.83 (27.40) | 0.080 | 0.573 |
|  | **Emotions subscale** | 40.91 (29.33) | 61.07 (26.22) | 63.68 (26.99) | 71.26 (26.04) | 0.045* | 0.326 |
|  | **Functioning subscale** | 22.12 (22.57) | 30.79 (25.26) | 51.57 (29.37) | 63.10 (29.34) | 0.161 | **1.153** |
| **EQ VAS (0-100)** | | 82.82 (13.25) | 74.41 (15.23) | 67.31 (19.35) | 59.00 (22.85) | 0.087 | 0.625 |
| **EQ-5D-5L (-0.848-1)** | | 0.93 (0.09) | 0.90 (0.09) | 0.82 (0.20) | 0.63 (0.39) | 0.099 | 0.708 |

‘Since COVID-19’ refers to after March 11, 2020.

*p*<0.05 for all groups except for one subgroup (Skindex-16 Emotions subscale-IGA. *p*-value was 0.071). Bolded relative efficiency values indicate that the measure is more efficient than DLQI at discriminating between known severity groups.

DLQI = Dermatology Life Quality Index; DLQI-R = Dermatology Life Quality Index Relevant; EASI = Eczema Area and Severity Index; IGA = Investigator Global Assessment; oSCORAD = Objective component of Scoring Atopic Dermatitis; VAS = Visual Analogue Scale.

References

1. Herdman M, Gudex C, Lloyd A, et al. Development and preliminary testing of the new five-level version of EQ-5D (EQ-5D-5L). *Qual Life Res*. 2011;20(10):1727-36. doi:10.1007/s11136-011-9903-x

2. Rencz F, Brodszky V, Gulácsi L, et al. Parallel Valuation of the EQ-5D-3L and EQ-5D-5L by Time Trade-Off in Hungary. *Value Health*. 2020;23(9):1235-1245. doi:10.1016/j.jval.2020.03.019

3. Finlay AY, Khan GK. Dermatology Life Quality Index (DLQI)--a simple practical measure for routine clinical use. *Clin Exp Dermatol*. 1994;19(3):210-6. doi:10.1111/j.1365-2230.1994.tb01167.x

4. Rencz F, Szabó Á, Brodszky V. Questionnaire Modifications and Alternative Scoring Methods of the Dermatology Life Quality Index: A Systematic Review. *Value Health*. 2021;24(8):1158-1171. doi:10.1016/j.jval.2021.02.006

5. Rencz F, Gergely LH, Wikonkál N, et al. Dermatology Life Quality Index (DLQI) score bands are applicable to DLQI-Relevant (DLQI-R) scoring. *J Eur Acad Dermatol Venereol*. 2020;34(9):e484-e486. doi:10.1111/jdv.16398

6. Rencz F, Gulácsi L, Péntek M, et al. Proposal of a new scoring formula for the Dermatology Life Quality Index in psoriasis. *Br J Dermatol*. 2018;179(5):1102-1108. doi:10.1111/bjd.16927

7. Chren MM, Lasek RJ, Sahay AP, Sands LP. Measurement properties of Skindex-16: a brief quality-of-life measure for patients with skin diseases. *J Cutan Med Surg*. 2001;5(2):105-10. doi:10.1007/bf02737863

8. Eichenfield LF, Lucky AW, Boguniewicz M, et al. Safety and efficacy of pimecrolimus (ASM 981) cream 1% in the treatment of mild and moderate atopic dermatitis in children and adolescents. *J Am Acad Dermatol*. 2002;46(4):495-504. doi:10.1067/mjd.2002.122187

9. Hanifin JM, Thurston M, Omoto M, Cherill R, Tofte SJ, Graeber M. The eczema area and severity index (EASI): assessment of reliability in atopic dermatitis. EASI Evaluator Group. *Exp Dermatol*. 2001;10(1):11-8. doi:10.1034/j.1600-0625.2001.100102.x

10. Stalder JF, Taïeb A, Atherton DJ, et al. Severity scoring of atopic dermatitis: the SCORAD index. Consensus Report of the European Task Force on Atopic Dermatitis. *Dermatology*. 1993;186(1):23-31. doi:10.1159/000247298

11. Kunz B, Oranje AP, Labrèze L, Stalder JF, Ring J, Taïeb A. Clinical validation and guidelines for the SCORAD index: consensus report of the European Task Force on Atopic Dermatitis. *Dermatology*. 1997;195(1):10-9. doi:10.1159/000245677
